# HIV-1 p24^gag^ Derived Conserved Element DNA Vaccine Increases the Breadth of Immune Response in Mice

**DOI:** 10.1371/journal.pone.0060245

**Published:** 2013-03-28

**Authors:** Viraj Kulkarni, Margherita Rosati, Antonio Valentin, Brunda Ganneru, Ashish K. Singh, Jian Yan, Morgane Rolland, Candido Alicea, Rachel Kelly Beach, Gen-Mu Zhang, Sylvie Le Gall, Kate E. Broderick, Niranjan Y. Sardesai, David Heckerman, Beatriz Mothe, Christian Brander, David B. Weiner, James I. Mullins, George N. Pavlakis, Barbara K. Felber

**Affiliations:** 1 Human Retrovirus Pathogenesis Section, Center for Cancer Research, Frederick National Laboratory for Cancer Research, Frederick, Maryland, United States of America; 2 Human Retrovirus Section, Vaccine Branch, Center for Cancer Research, Frederick National Laboratory for Cancer Research, Frederick, Maryland, United States of America; 3 University of Pennsylvania, Philadelphia, Pennsylvania, United States of America; 4 Departments of Microbiology Medicine and Laboratory Medicine, University of Washington, Seattle, Washington, United States of America; 5 Ragon Institute of MGH, MIT and Harvard, Boston, Massachusetts, United States of America; 6 Inovio Pharmaceuticals, Inc., Blue Bell, Pennsylvania, United States of America; 7 Microsoft Research, Redmond, Washington, United States of America; 8 IrsiCaixa AIDS Research Institute-HIVACAT, Autonomous University of Barcelona, Barcelona, Spain; 9 Institucio Catalana de Recerca i Estudis Avancats (ICREA), Barcelona, Spain; University of Catania, Italy

## Abstract

Viral diversity is considered a major impediment to the development of an effective HIV-1 vaccine. Despite this diversity, certain protein segments are nearly invariant across the known HIV-1 Group M sequences. We developed immunogens based on the highly conserved elements from the p24^gag^ region according to two principles: the immunogen must (i) include strictly conserved elements of the virus that cannot mutate readily, and (ii) exclude both HIV regions capable of mutating without limiting virus viability, and also immunodominant epitopes located in variable regions. We engineered two HIV-1 p24^gag^ DNA immunogens that express 7 highly Conserved Elements (CE) of 12–24 amino acids in length and differ by only 1 amino acid in each CE (‘toggle site’), together covering >99% of the HIV-1 Group M sequences. Altering intracellular trafficking of the immunogens changed protein localization, stability, and also the nature of elicited immune responses. Immunization of C57BL/6 mice with p55^gag^ DNA induced poor, CD4^+^ mediated cellular responses, to only 2 of the 7 CE; in contrast, vaccination with p24CE DNA induced cross-clade reactive, robust T cell responses to 4 of the 7 CE. The responses were multifunctional and composed of both CD4^+^ and CD8^+^ T cells with mature cytotoxic phenotype. These findings provide a method to increase immune response to universally conserved Gag epitopes, using the p24CE immunogen. p24CE DNA vaccination induced humoral immune responses similar in magnitude to those induced by p55^gag^, which recognize the virus encoded p24^gag^ protein. The inclusion of DNA immunogens composed of conserved elements is a promising vaccine strategy to induce broader immunity by CD4^+^ and CD8^+^ T cells to additional regions of Gag compared to vaccination with p55^gag^ DNA, achieving maximal cross-clade reactive cellular and humoral responses.

## Introduction

The extensive variability of HIV is a major stumbling block in vaccine design, since successful vaccines must protect against widely diverse virus strains. The plasticity of the HIV genome allows for a vast number of mutations that can escape immune responses while preserving protein function. As a result, there is currently no candidate HIV vaccine that predictably elicits broad, durable and protective immune responses. To maximize immunologic breadth, several alternative approaches are being explored, including the use of consensus, ancestral or center-of-tree immunogens, multiple strains and mosaic immunogens, immunogens consisting of known epitopes from the database, algorithm-selected epitopes or a selection of the most conserved epitopes from different clades as one chimera [1]–[19]. It may not be possible to vaccinate with all of the viral antigenic diversity required to block viable or transitional escape forms of the virus [20], [21] because, to be practical, a vaccine should preferably consist of as few components as possible. Additional concerns have been raised against the use of multiple variable epitopes. First, using a multi-strain or mosaic-like approach may result in the expansion of T cell reactivity to highly immunodominant epitopes (i.e., having hierarchical preference for one epitope of the vaccine over another, magnified by its presentation in different variant forms). Second, variable epitopes may divert responses (i.e., suppression of some responses by others) towards non-protective epitopes [22], [23]. Except for individuals with “protective HLA alleles” such and HLA-B27 and HLA-B57 [24]–[28], recognition of immunodominant epitopes generally does not result in virologic containment *in vivo* (e.g., low viral loads) [29], [30]. Immunodominant but variable epitopes readily undergo immunologic escape by accumulating mutations that do not impair viral fitness [31]–[34], and thus, they may not contribute substantially to a vaccine's protective responses. Variable epitopes may therefore serve as immunodominant “decoys” that could usurp immune reactivity and potentially preclude the induction of responses against protective epitopes [35].

Our approach to the development of protective immunogens [35]–[37] derives from a conceptual coalescence of recent findings: Viral proteins recover ancestral amino acid (AA) states when transmitted to a new host [38], essentially recovering a more fit state in the absence of the specific immune responses found in the previous host [39]–[41]; changes in conserved AA of viral proteins can debilitate or destroy virus viability [42]–[45]; high avidity CTLs recognizing some conserved viral epitopes are present in controllers and long-term non-progressors (LTNP) [37], [46]; CTL responses against specific viral proteins (e.g., Gag) are associated with control of viremia [32], [47]–[50]; immunodominance obscures or prevents reactivity against other potentially more protective epitopes [51]; and some AA segments in HIV proteins are conserved throughout a given HIV-1 subtype, the entire M group of HIV-1, and even in some instances in HIV-2 and SIV [35], [52]. These observations suggest that persistent immune targeting of viral conserved elements while excluding variable immunodominant epitopes, thereby avoiding the potential negative effects of directing T cell responses to “decoy” epitopes, may lead to protective immunity [35], [52]. In fact, it was demonstrated that the presence of dominant epitopes effectively suppressed the subdominant responses and that this effect could be modulated [53], [54]. Thus, if the goal of the vaccine is to induce responses to subdominant regions, then the most frequently targeted, potential “decoy” epitopes should be omitted from the immunogen sequence.

Several studies have shown that Gag-specific T cell responses correlate with control of viremia in clade B and C infected individuals [31], [32], [55], [56]. In addition, a cross-sectional *ex vivo* study, using samples from individuals with different HLA haplotypes, identified p24^gag^ T cell responses of high functional avidity and broad variant recognition associated with relative control of HIV infection [37]. Our conserved element approach to design an HIV vaccine immunogen calls for inclusion of virtually invariant amino acid strings of viral proteins of at least 8 AA in length (representing the minimal length of a CTL epitope), as these are more likely to be required for viral fitness. Additionally, all variable regions of the virus that, by virtue of their structural plasticity, might contain immunogenic sites capable of existing in alternative functional states (potential immunodominant “decoys”) were excluded. Database exploration identified 7 segments of p24^gag^ that fit our criteria for inclusion in a novel Conserved Element immunogen (p24CE). In the present proof-of-concept study, we used DNA vectors expressing two variants of the highly conserved p24CE immunogens to characterize cellular and humoral responses in comparison to full-length p55^gag^ immunogens in vaccinated mice.

## Results

### Conserved Element DNA vaccines

**Figure 1:**
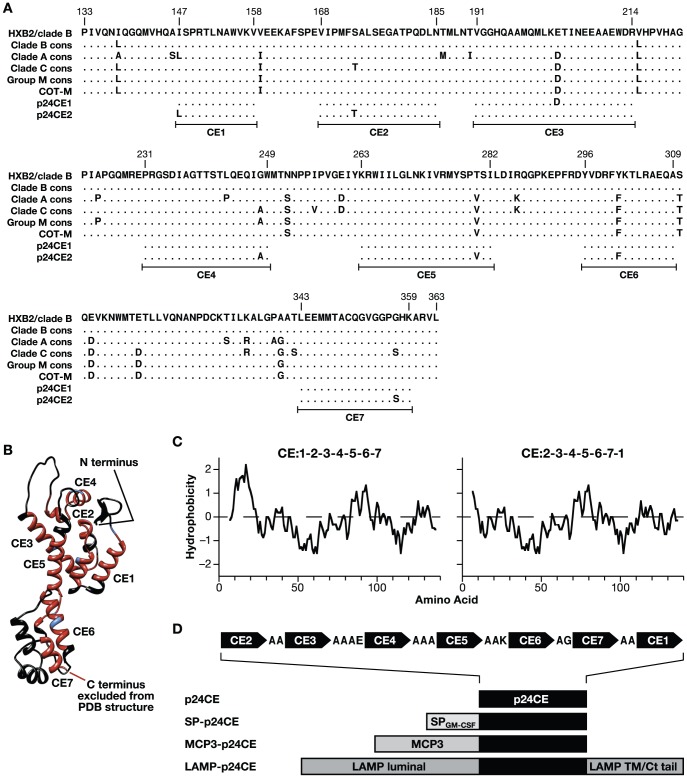
Design of the p24CE DNA vaccine. (**A**) Alignment of the HXB2 p24^gag^ protein sequences with the consensus clades A, B and C and the Group M consensus, the Group M Center-of-Tree (COT-M) and the 7 CE included in p24CE1 and p24CE2. The ‘toggle’ amino acid differences between the CE1 and CE2 sequences are indicated. (**B**) Localization of CE within the hexameric p24^gag^ structure. The p24^gag^ structure is modified from Pornillos et al. [57] and shows the location of CE1-CE7 (red), the toggle AA (blue) and the AA not included in the CE (black). The crystal structure of the hexamer was obtained from http://www.ebi.ac.uk/pdbsum/. (**C**) Kyte-Dolittle hydrophobicity plots for two different collinear arrangements of the CEs. (**D**) The p24CE (p24CE1 and p24CE2) proteins are composed of 7 CE arranged collinearly and linked via amino acid linkers. The secreted SP-p24CE contains the GM-CSF signal peptide. MCP3-p24CE is a fusion protein with the Monocyte chemoattractant protein 3 (MCP3) chemokine. LAMP-p24CE is a fusion with the lysosomal associated membrane protein 1 (LAMP-1).

A set of conserved elements (CE) was identified in p24^gag^ composed of amino acids (AA) highly conserved across the entire HIV-1 group M, as determined by using the Los Alamos HIV database (www.hiv.lanl.gov/) [35]. A refined list of 7 CE was selected based on several criteria (**Figure 1A**, see Materials and Methods): a minimum length of 8 AA; inclusion of specific epitopes that have been correlated with viral control (low viral loads) *in vivo*; and exclusion of epitopes associated with high viral loads. The selected CE span 12–24 AA each, and together a total of 124 AA, thus representing 54% of p24^gag^ sequence. The CE are highlighted on the p24^gag^ capsid ribbon structure [57], revealing that they encompass most of the extended coiled regions of the p24^gag^ protein **(** **Figure 1B**).

We generated multiple DNA-based vaccines in which the 7 CE were collinearly arranged (**Figure 1C**), and connected via short linker sequences (**Figure 1D**), designed for efficient proteolytic cleavage [58], [59]. Proteolytic processing of CE peptides *in vitro* revealed production of optimal epitopes or slightly extended optimal epitopes from 6 of the 7 CE segments (S. Le Gall, in preparation). Therefore, the CE immunogen is predicted to be able to present a significant number of native T cell epitopes after expression. For optimal arrangement of the different segments within the p24CE immunogens, the hydrophobicity of individual CE was taken into consideration (**Figure 1C**). To avoid the strongly hydrophobic N-terminus in the arrangement CE1-2-3-4-5-6-7 (left panel), which could impact the intracellular trafficking of the protein, the CE1 peptide was placed at the C-terminus (right panel).

The majority of the AA included in the p24CE immunogens are essentially invariant since they are found in >98% of HIV isolates. The length of p24CE was expanded by including some less well-conserved AA (‘toggle’ sites), thus expressing additional potentially immunogenic regions. This allowed the extension of the 7 CE to the length of 12–24 AA, as mentioned above, and led to two p24CE sequences differing by 7 AA, one in each CE (**Figure 1A**), named p24CE1 and p24CE2. These two sequences cover >99% of all known HIV-1 group M sequences. The p24CE1 and p24CE2 sequences were RNA/codon optimized [60]–[63] to maximize mRNA processing, transport, stability and translation (see Material and Methods). The p24CE coding regions were cloned into the pCMVkan vaccine vector (p24CE; **Figure 1D**). Additional expression plasmids were generated in order to alter the intracellular trafficking and processing of the p24CE proteins. Plasmids SP-p24CE1 and SP-p24CE2 contain the GM-CSF signal peptide at the N-terminus of p24CE to promote secretion of the p24CE proteins. Plasmids MCP3-p24CE1 and MCP3-p24CE2 express fusion proteins with the monocyte chemoattractant protein 3 (MCP-3) chemokine, previously shown to stabilize the encoded protein and to enhance trafficking to antigen presenting cells [64]–[66]. Plasmids LAMP-p24CE1 and LAMP-p24CE2 express fusion proteins with the human lysosomal associated membrane protein 1 (LAMP-1). Fusion of Gag to LAMP was previously shown to direct it to the lysosomal compartment and to facilitate access to the MHC class II pathway as well as to the extracellular compartment [67]–[71].

### Expression of the p24CE proteins in human cells

**Figure 2:**
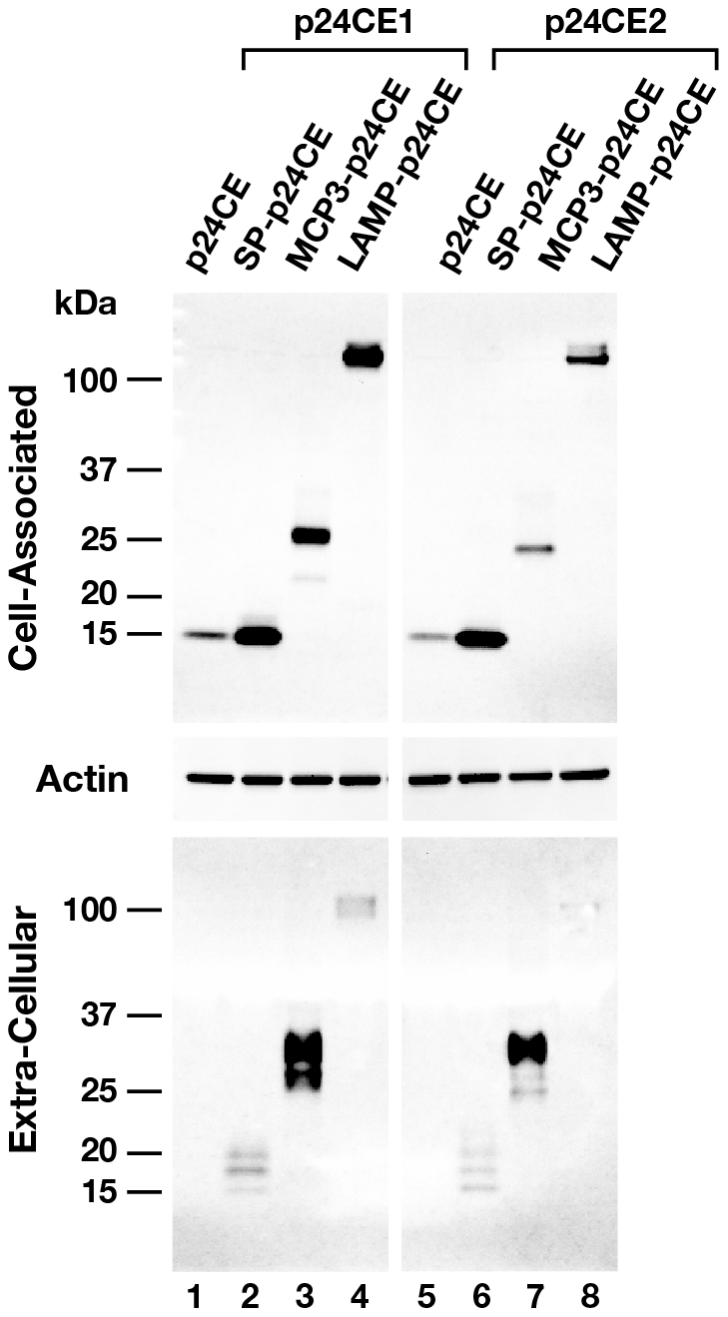
Expression of the p24CE plasmids upon transient transfection in cultured cells. Plasmid DNA (1 µg) expressing different variants of either p24CE1 (left panel) or p24CE2 (right panel) proteins were transfected in HEK293 cells. The cultures were harvested 24 hrs later and proteins from equal amounts (1/250) from the cell-associated (top panel) and extra-cellular (bottom panel) fractions were resolved on a 12% NuPAGE Bis-Tris gel and analyzed by Western immunoblot using a goat anti-p24^gag^ antiserum and visualized using enhanced ECL. The membrane containing the cell-associated fractions was also probed with anti-human pan actin antibody to control for equal loading of the samples.

The expression of the p24CE vectors shown in Figure 1D was evaluated by Western immunoblots using cell extracts and supernatants from transiently transfected HEK293 cells (**Figure 2**). To control for equal loading, the membrane containing the cell-associated samples were probed with an antibody against human beta actin as internal control (middle panel) demonstrating that similar amounts of proteins were loaded into each lane which validates our conclusions regarding the stability and different distribution of the proteins encoded by the different transfected plasmids (see below). Very low levels of the p24CE1 and p24CE2 proteins were detected in the cell-associated fractions (lanes 1 and 5, respectively), and no proteins were found in the extracellular compartment, indicating that the p24CE proteins were unstable. We also noted that p24CE2, differing only by 7 of the 124 AA from p24CE1, produced an even less stable antigen. The presence of the GM-CSF signal peptide (SP) greatly increased the levels of both p24CE proteins (lanes 2 and 6) in both the cell-associated and the extracellular fractions. These data indicate that the signal peptide altered the trafficking of the p24CE proteins, and promoted increase in stability and secretion. We noted the presence of additional bands of the secreted p24CE proteins, likely due to posttranslational modifications related to the altered cellular trafficking (compare lane 2 and lane 1; lane 6 to lane 5). The MCP3-p24CE (lanes 3 and 7) and LAMP-p24CE fusion proteins (lanes 4 and 8) were also readily detectable, and thus these fusions greatly stabilized the p24CE proteins. MCP3-p24CE localization in the extracellular fraction (lanes 3 and 7) as several bands, was similar to our previous report on a MCP3-Gag fusion protein [64]. The LAMP-p24CE proteins accumulated primarily in the cell-associated fraction (lanes 4 and 8), although some protein could also be found in the extracellular fraction, as we previously observed for the LAMP-p55^gag^ protein [70]. These data showed that altering the trafficking of the p24CE proteins, by adding the GM-CSF signal peptide or upon fusion to the MCP3 or LAMP molecules, enhanced the stability and modulated the trafficking of p24CE proteins.

### Vaccination with p24CE induces CE-specific cellular immune responses in C57BL/6 mice

**Figure 3:**
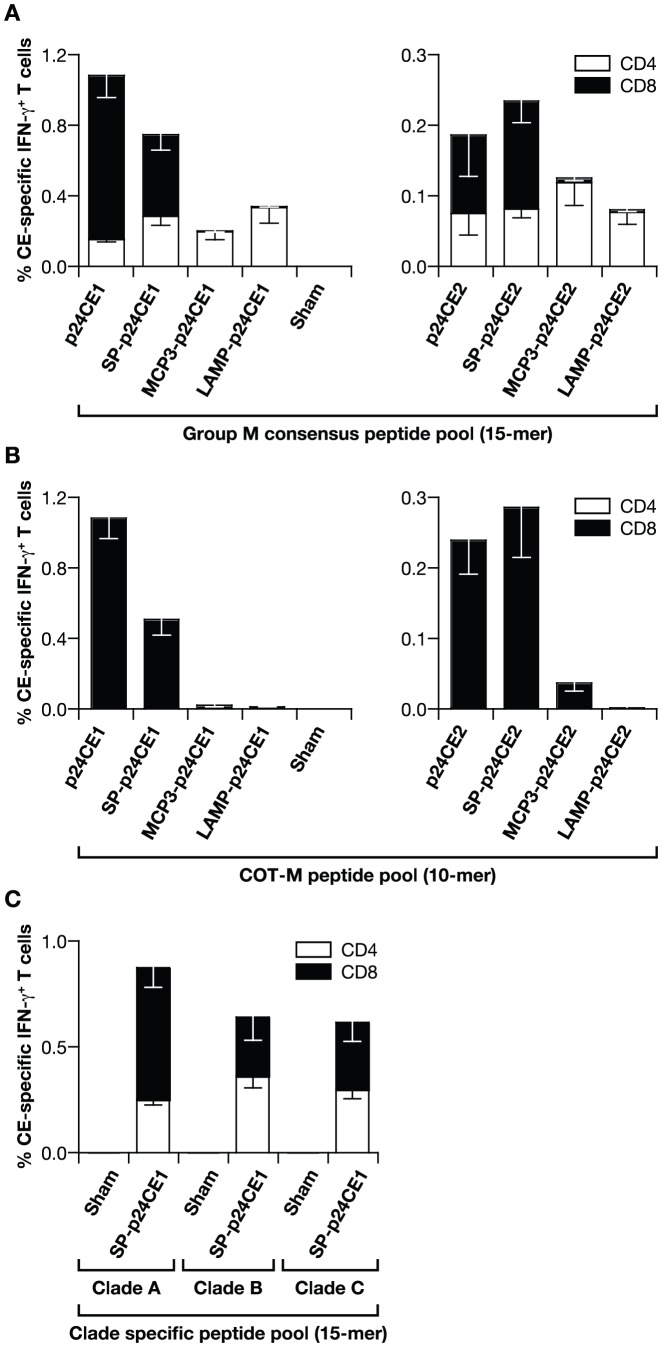
Cellular responses in p24CE DNA vaccinated C57BL/6 mice. Mice were vaccinated using *in vivo* EP with 20 µg of the indicated p24CE1 (left panel) or p24CE2 (right panel) DNA plasmids (**A, B**) or with SP-p24CE1 DNA (**C**). Splenocytes from individual animals were stimulated (**A**) with the Group M Consensus peptide pool (15-mer peptides overlapping by 11 AA), (**B**) with the COT-M peptide pool (10-mers overlapping by 9 AA) consisting of the matching peptides of p24CE1 and p24CE2 proteins, and (**C**) with peptide pools representing the clade A, B, and C p55^gag^ sequences (15-mer peptides overlapping by 11 AA), as described in Materials and Methods. The frequency of CE-specific IFN-γ producing CD4^+^ (open bars) and CD8^+^ (filled bars) T cells was determined by polychromatic flow cytometry. The mean and SEM are shown. Three experiments were performed and data from a representative experiment are shown.

We next evaluated the immunogenicity of different p24CE proteins after DNA vaccination of C57BL/6 mice. Groups of mice (N = 5) were vaccinated twice (0 and 4 weeks) with the indicated p24CE plasmids or sham plasmid DNA, as negative control, by intramuscular injection followed by *in vivo* electroporation (EP). Two weeks after the last vaccination (week 6), the mice were sacrificed and the presence of CE-specific cellular responses was determined by polychromatic flow cytometry. Splenocytes from the individual animals from each of the vaccine groups and the sham DNA inoculated negative control group were stimulated with a Group M consensus Gag peptide pool (15-mer peptides overlapping by 11 AA) (**Figure 3A**) or with a COT-M peptide pool (10-mer overlapping by 9 AA) consisting of both p24CE1 and p24CE2 sequences (**Figure 3B**). The use of the 15-mer peptide pool allowed for the detection of both CD4^+^ and CD8^+^ T cell responses, whereas the 10-mer peptide pool favors mainly CD8^+^ T cell responses. Vaccination with plasmids expressing p24CE or the secreted p24CE (SP-p24CE) proteins induced both CE-specific CD4^+^ and CD8^+^ T cell immune responses (**Figure 3A**). In contrast, vaccination with the p24CE fusion proteins, MCP3-p24CE or LAMP-p24CE, elicited CE-specific responses that were almost exclusively mediated by CD4^+^ T cells. In agreement with these results, splenocyte stimulation with 10-mer peptide pools, which are mainly associated with MHC class I antigens, induced very low responses in MCP3-p24CE DNA vaccinated mice and no responses in the LAMP-p24CE immunized mice, which verified the previous conclusions (**Figure 3B**). We hypothesize that altered intracellular trafficking of the p24CE fusion antigens could be responsible for the distinct preference for CD4^+^ or CD8^+^ T cell responses. Responses elicited by p24CE1 proteins were in general higher than those induced by p24CE2 (**Figure 3A and 3B**, note the different scales for p24CE1 and p24CE2), likely reflecting the higher expression of p24CE as indicated by the transient transfection experiments (see **Figure 2**). As expected, no cellular responses were found in splenocytes from sham DNA vaccinated mice.

The cross-reactivity of the induced responses was analyzed using peptide pools representing different HIV-1 clades (A, B, and C; see also **Figure 1A**). SP-p24CE1 DNA vaccination induced cross-clade reactive CD4^+^ and CD8^+^ cellular responses, which were similar in magnitude to those obtained with the Group M peptide pool (**Figure 3C**). Cross-clade reactivity was also obtained upon vaccination with the other p24CE plasmids (data not shown). In contrast, splenocytes from mice immunized with sham DNA failed to recognize peptides from any of the three clade-specific peptide pools.

### Fine specificity of CE-specific T cell responses from vaccinated C57BL/6 mice

**Figure 4:**
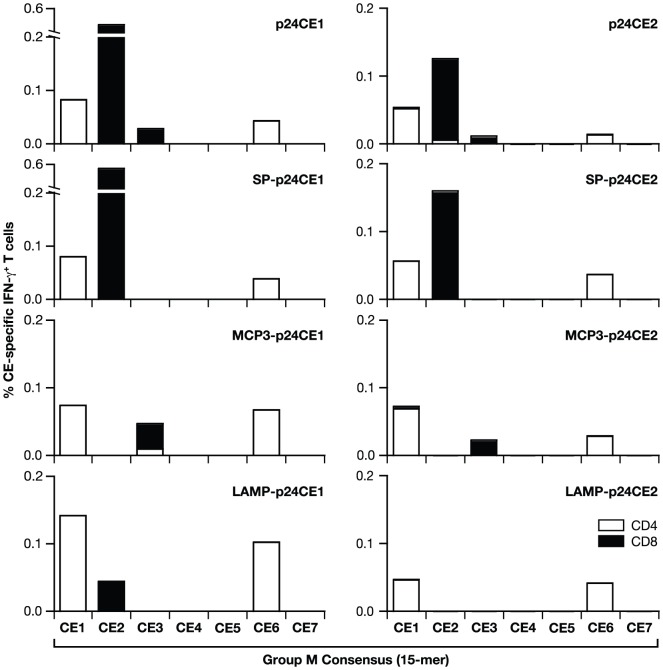
Mapping of the p24CE-induced cellular immune responses. Pooled splenocytes from C57BL/6 mice (N = 5) vaccinated with the indicated p24CE1 (left panels) or p24CE2 (right panels) DNAs were stimulated with the Group M Consensus peptide pools (15-mers overlapping by 11 AA) spanning the individual CEs. The frequency of CE-specific IFN-γ producing T cells was measured. CD4^+^ (open bars) and CD8^+^ (filled bars) Gag-specific T cells are shown.

Next, we assessed the distribution of the p24CE-induced cellular responses among the different CE (**Figure 4**). Pooled splenocytes from the DNA vaccinated C57BL/6 mice (N = 5/group) were stimulated with Group M consensus peptide pools (15-mer) encompassing the 7 individual CE. Polychromatic flow cytometry was used to determine the frequency of the CE-specific IFN-γ producing T cells and to discriminate between CD4^+^ and CD8^+^ T cell responses. Immunization with the different p24CE1 (left panels) and p24CE2 (right panels) plasmid DNAs induced cellular responses to CE1 and CE6, which were mediated almost exclusively by CD4^+^ T cells. Interestingly, mice immunized with plasmids encoding the native p24CE protein (p24CE and SP-p24CE) developed also high CD8^+^ mediated cellular responses to CE2. These data are in agreement with the cellular localization of the encoded proteins: the native p24CE protein remains mainly intracellular, while the SP-p24CE and MCP3-p24CE fusion are actively secreted and the LAMP-p24CE associates with the MHC class II compartment. Low levels of CD8^+^ T cell responses to CE3 were also identified upon immunization with the p24CE and the MCP3-p24CE plasmids. In conclusion, the p24CE proteins induced responses to 4 of the 7 CE (CE1, CE2, CE3, CE6) in mice, although these responses were generally lower in animals immunized with the p24CE2 plasmids, demonstrating that vaccination induced broad CD4^+^ and CD8^+^ T cell responses.

### p24CE induces broader immune responses than the full-length p55^gag^

**Figure 5:**
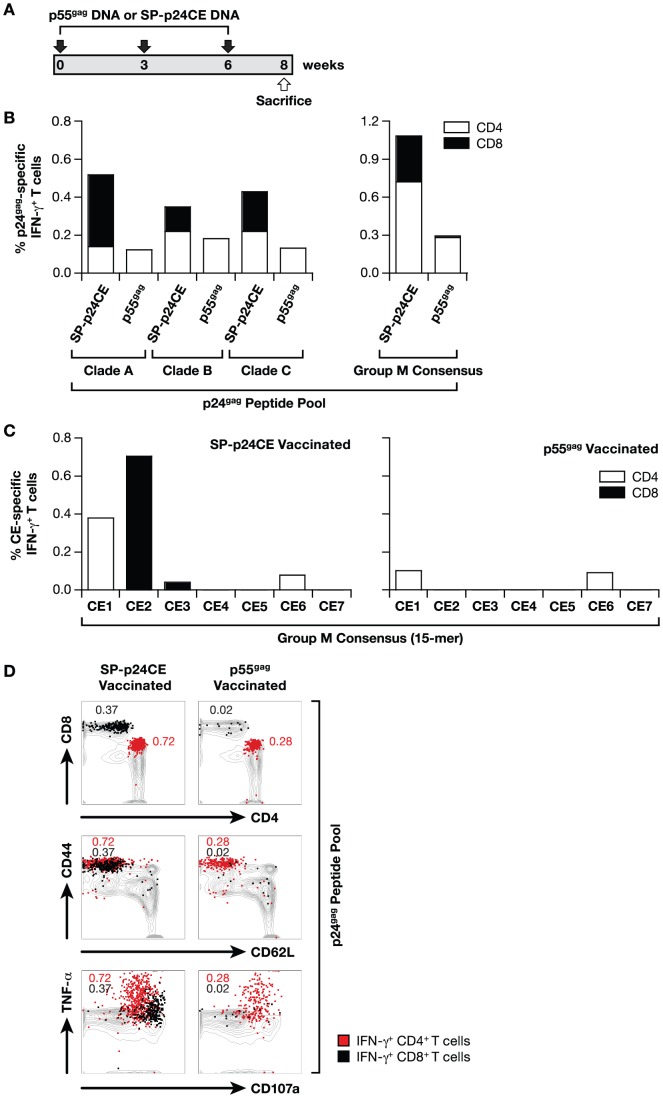
Phenotypic and functional analysis of T cell responses generated by p55^gag^ and p24CE DNA vaccination. **(A)** Mice (N = 5/group) were vaccinated 3 times (week 0, 3 and 6) with 20 µg of a plasmid expressing HXB2 p55^gag^ (clade B) or 20 µg of a mixture of plasmids expressing SP-p24CE1 and SP-p24CE2. The mice were sacrificed 2 weeks after the last immunization. Three independent experiments were performed and a representative experiment is shown. (**B**) Pooled splenocytes were stimulated with Clade A, B or C peptide pools (15-mers) spanning the p24^gag^ region (left panel) and the Group M consensus peptide pool (right panel). The frequency of the CD4^+^ (open bars) and CD8^+^ (filled bars) p24^gag^-specific IFN-γ producing T cells was determined. (**C**) The splenocytes from the SP-p24CE (left panel) and p55^gag^ (right panel) DNA vaccinated mice were stimulated with peptide pools specific for the individual CEs. The frequency of the CD4^+^ (open bars) and CD8^+^ (filled bars) CE-specific IFN-γ producing T cells was determined. (**D**) Plot overlays show the phenotypic and functional characterization of the antigen-specific T cells induced by SP-p24CE (left panels) and p55^gag^ (right panels) DNA vaccines upon stimulation with p24^gag^–specific peptide pool. Total T cells recovered from the spleen are shown as grey contours, and the antigen-specific IFN-γ^+^ T cells are overlaid as red (CD4^+^ T cells) or black (CD8^+^ T cells) dots. The plots show the CD4/CD8 distribution (top panel), memory phenotype as determined by CD44/CD62L staining (middle panel) and TNF-α/CD107a expression (bottom panel) among the T cells from vaccinated mice. The frequency of CD4 (red) and CD8 (black) IFN-γ T lymphocytes is shown.

We compared the immune responses to the individual CE upon vaccination with a p55^gag^ plasmid DNA or with a mixture of SP-p24CE1 and SP-p24CE2 DNAs. The mice (N = 5/group) received 3 vaccinations (week 0, 3 and 6) and were sacrificed at week 8 (**Figure 5A**). Vaccine-induced T cell responses were analyzed from pooled splenocytes (**Figure 5B**) stimulated with 15-mer peptide pools specific for p24^gag^ of clade A, B, or C (left panel) and the group M consensus (right panel). The overall responses induced by the p55^gag^ immunogen were lower than those obtained by the p24CE immunogen, and remarkably, lacked CD8^+^-specific T cells. Using peptide pools spanning the individual CE (**Figure 5C**) showed that p55^gag^ DNA elicited low responses to CE1 and CE6 only (right panel), mediated exclusively by CD4^+^ T cells. In contrast, vaccination with SP-p24CE DNA mixture elicited higher responses towards several CE (CE1, CE2 CE3 and CE6), as also expected from the data shown in Figure 4.

We also evaluated the quality of the cellular immune responses elicited by the different immunogens (**Figure 5D**) using the p24^gag^ peptide pool followed by intracellular cytokine staining and polychromatic flow cytometry. Vaccination with p55^gag^ DNA induced primarily CD4^+^ (red) T cell responses, while p24CE vaccination induced both CD4^+^ (red) (CE1 and CE6) and CD8^+^ (black) (CE2 and CE3) T cell responses (**Figures 5B and 5C**, top panel). Both immunogens induced effector memory T cells (CD44^hi^ and CD62L^neg^) (Figure 5C, middle panel), which were mainly CD4^+^ (0.28% of total T cells) in mice vaccinated with p55^gag^ DNA, and both CD4^+^ (0.72% of total T cells) and CD8^+^ (0.37% of total T cells) in the mice vaccinated with the SP-p24CE DNA. Further analyses revealed that antigen-specific IFN-γ^+^ T cells produced TNF-α and expressed CD107a on the surface upon stimulation with antigen, indicating induction of cytotoxic T cells (**Figure 5D**, bottom panel). We also noted that the CD8^+^ T cells (black), induced only by SP-p24CE DNA vaccination, expressed higher levels of CD107a and lower levels of TNF-α than the CD4^+^ T cells (red), a phenotype consistent with the degranulation associated with CTL activity. Collectively our results show that the p24CE vaccine increased breadth and magnitude of cellular responses to p24^gag^ region in DNA vaccinated mice, by inducing robust responses to several of the highly conserved elements, and that the responses are multifunctional, a desired feature for an effective HIV vaccine.

### Vaccination with p24CE induces cross-clade reactive humoral immune responses

**Figure 6:**
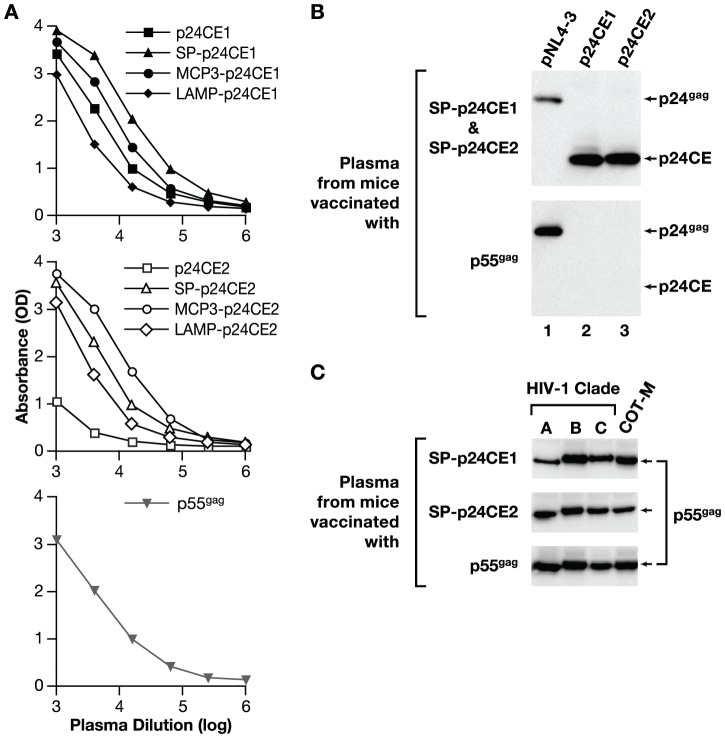
Humoral immune responses in p24CE DNA vaccinated mice. (**A**) Anti-HIV-1 p24^gag^ antibodies were measured in plasma from p24CE and p55^gag^ DNA vaccinated C57BL/6 mice by a standard clade B p24^gag^ ELISA. The graphs show absorbance (optical density, OD) and pooled plasma samples dilutions from mice vaccinated with the different p24CE1 plasmids (top panel), p24CE2 plasmids (middle panel), or p55^gag^ DNA (bottom panel). (**B**) Humoral responses induced upon SP-p24CE or p55^gag^ DNA vaccination in mice were analyzed by Western immunoblot assays. The membranes contain p24^gag^ protein collected from supernatants of HEK293 cells transfected with 5 µg of the infectious molecular clone pNL4-3 (lane 1) or the p24CE proteins collected from the cell-associated fractions of cells transfected with SP-p24CE1 and SP-p24CE2 plasmids (lanes 2 and 3, respectively). The membranes were probed with plasma (1∶5000 dilution) from mice vaccinated with a mixture of SP-p24CE1&2 DNAs (top panel) or p55^gag^ DNA (bottom panel) followed by anti-mouse IgG-HRP labeled antibody and visualized by ECL. (**C**) Detection of humoral responses to full-length p55^gag^ in mice vaccinated with p24CE or p55^gag^ DNA by Western immunoblot assay. The p55^gag^ proteins were obtained from HEK293 cells transfected with 0.5 µg of RNA/codon optimized plasmids expressing unprocessed p55^gag^ from clades A, B and C or COT-M, respectively. The proteins were resolved on 10% NuPAGE Bis-Tris gels, and the membranes were probed with plasma (dilution 1∶200) from mice immunized with DNAs expressing the secreted p24CE proteins SP-p24CE1 (top panel), SP-p24CE2 (middle panel) and p55^gag^ (bottom panel).

We next examined the induction of humoral immune responses using pooled plasma samples from p24CE DNA vaccinated mice (N = 5/group) by an ELISA measuring clade B p24^gag^ responses (**Figure 6A**). The different p24CE antigens readily induced high levels of humoral responses with titers similar or greater than those achieved in p55^gag^ DNA vaccinated mice (except p24CE2, middle panel). Vaccination with p24CE DNA induced antibodies to both the p24CE proteins (**Figure 6B**, top panel, lanes 2 and 3) as well as to the processed p24^gag^ protein (lane 1). In contrast, the antibodies induced by p55^gag^ DNA vaccination readily detected p24^gag^ (Figure 6B, bottom panel, lane 1), but failed to recognize the p24CE proteins (lanes 2 and 3). Thus, similar to the cellular immune responses (see **Figure 5**), the antibodies elicited upon vaccination with full-length p55^gag^ DNA were unable to recognize the conserved elements.

We also examined the cross-clade reactivity of these responses by Western immunoblot analysis (**Figure 6C**). Membranes containing p55^gag^ proteins from consensus clades A and C, clade B (HXB2) and COT-M, obtained from transiently transfected cells, were probed with pooled plasma samples from mice vaccinated with plasmids expressing SP-p24CE1, SP-p24CE2 or p55^gag^. The Western immunoblot assays showed that the antibodies induced by the p24CE and p55^gag^ DNA vaccinated mice detect the different p55^gag^ proteins. These data suggest that similar to p55^gag^, p24CE vaccinated mice induce cross-clade reactive antibodies.

Together, these data show that p24CE DNA vaccination induced strong humoral (**Figure 6**) and cellular (**Figure 4**) immune responses to the highly conserved elements in p24^gag^, and that CE segments are not or only poorly immunogenic when expressed as part of the complete p55^gag^ in DNA vaccinated C57BL/6 mice.

## Discussion

The present work is a proof-of-principle study to evaluate whether a DNA vaccine expressing 7 selected highly Conserved Elements within HIV-1 p24^gag^ can be produced and whether this DNA vaccine is immunogenic in comparison to DNA-encoded full-length native p55^gag^. We have previously demonstrated that individuals chronically infected with HIV-1 develop cellular immune responses specific for the peptides encoded by the 7 conserved elements described in this work [37]. Furthermore, we found that the breadth, magnitude and avidity of these cellular responses to some CE were significantly higher among patients able to control HIV-1 infection, which suggests that responses against these conserved regions are clinically relevant [37].

Starting from our understanding of the rules for robust gene expression, and to avoid the escape potential of HIV, we have constructed optimized DNA vectors that express maximal levels of new artificial immunogens based on highly conserved elements of the p24^gag^ region. This vaccine design is based on two principles, (i) the immunogen must include critical and highly conserved elements of the virus that cannot mutate without a severe loss in viability, and (ii) the immunogen must exclude HIV epitopes that are capable of mutating without significantly affecting viral fitness. The former may induce responses against a large number of HIV isolates, and the latter avoids immunodominant competition from variable regions, which may render ineffective the vaccine-induced immune response. Not only expression, but also the stability and presentation of the artificial antigens encoded by the DNA vectors were optimized. To this end, different fusion constructs were designed. We previously noted that either addition of a signal peptide or fusion to either MCP3 or LAMP were beneficial for protein expression [64], [66], [72], and found that these modifications also stabilize the p24CE proteins.

To maximize stimulation of CD4^+^ cells in addition to CD8^+^ T cells, we designed secreted immunogens. The p24CE antigen linked to the signal peptide of GM-CSF was expressed at high levels and also produced both CD4^+^ and CD8^+^ antigen-specific T cells as well as good antibody titers. In contrast, p24CE proteins fused to MCP3 or LAMP directed the development of mostly CD4^+^ T cell responses. These studies show that it is possible to manipulate many properties of an antigen, altering the immune response in predictable ways. Although CD8^+^ T cell responses have been linked to control of viremia, we have also reported that cytotoxic CD4^+^ T cell responses contribute to viral control [73]. The p24CE vaccine induced both CD4^+^ (CE1 and CE6) as well as CD8^+^ (CE2 and CE3) specific T cell responses in the C57BL/6 mice; these CD8^+^ T cells had the functional phenotype of mature CTLs and were absent in mice immunized with the DNA encoding p55^gag^. The molecules generated allow the selection of the most optimal combinations to achieve the best protective response for HIV prophylaxis. We have found that p24CE proteins are more immunogenic than the full-length Gag protein, expanding the quality of cellular responses to recruit CD8^+^ T cells with the functional properties of canonical CTLs in C57BL/6 mice. These findings suggest that peptides, containing the CE regions, which were produced from the full-length p55^gag^ protein were not recognized efficiently by the T cells. This could be due to poor antigen processing or presentation, or alternatively due to interference by immunodominant peptides from other regions within p55^gag^, which are able to divert or inhibit immune response. In addition, as shown in Figures 3 and 4, the trafficking of the protein greatly affected its immunogenicity, with the p24CE and SP-p24CE eliciting the highest and most balanced CD4 and CD8 responses. Selection of the most optimal p24CE protein induced higher and broader immunogenicity than p55^gag^. We have recently also shown that dendritic cells *in vitro* loaded with RNA encoding the p24CE described in this work are able to stimulate T cell responses when mixed with autologous PBMC from HIV patients or to induce de novo T cell responses in PBMC from healthy donors. The responses elicited by p24CE were usually as high as those by full-length Gag [36].

All the immunogenicity studies described in the present work were performed in C57BL/6 mice. In our experience, p55^gag^ DNA immunization using the Balb/c mouse model induces higher T cell responses, but those responses are almost exclusively directed towards a single immunodominant epitope, AMQMLKETI, which is present in our p24CE construct. Therefore, to avoid the restrictions imposed by this limited repertoire, we chose the C57BL/6 mouse model for the work described herein. We found very low primary immune responses to the CE regions after DNA vaccination using full-length p55^gag^. It will be of interest to further examine whether other vaccine modalities, i.e. recombinant viral vectors, expressing p55^gag^ are able to induce higher immune responses to the CE. To our knowledge, this study is the first comparative evaluation of immunity induced by a full-length immunogen and that induced by highly conserved elements from within the same protein. Our analysis points to the negative effect of regions outside of the defined conserved elements, which, it is important to note, are present in the full-length wild type as well as in the consensus and mosaic molecules as well as in the reported epitope immunogens. Thus, the use of the highly Conserved Element platform offers the advantage of focusing the immune responses to the invariable epitopes present in the viral proteome. Similar to the work described here, Letourneau et al. [11] previously demonstrated that a chimeric protein containing a string of several invariable regions from the HIV-1 proteome was immunogenic, but a direct comparison with the same sequences expressed within the natural proteins was not performed. In our study, we applied more stringent criteria to define conserved elements resulting in shorter peptide sequences (12–24 AA) that exclude adjacent more variable segments. In addition, our analysis of the immune responses was performed using peptide pools strictly confined to the conserved segments defined as immunogens and, therefore, the contribution to the T cell responses of putative artificial new epitopes created by the boundaries was completely excluded. In conclusion, we showed that the p24CE DNA vaccine induced broad cross-clade reactive cellular and humoral responses in vaccinated mice. We detected robust immune responses, including CD8^+^ T cells, to several CE upon p24CE DNA vaccination in mice, whereas only very poor (CD4^+^ only) or no responses to the CE were obtained by DNA vaccination with vectors expressing full-length p55^gag^. Thus, the inclusion of DNA vectors expressing the conserved elements is a promising vaccine strategy to induce broader immunity compared to vaccination with the p55^gag^ DNA alone. These results suggest further evaluation of the p24CE antigens in macaques.

## Materials and Methods

### p24^gag^ Conserved Elements selection

Using all HIV-1 M group p24^gag^ coding sequences available in the 2009 Los Alamos database, we identified sequences of at least 8 AA in length, in which all AA were conserved in at least 98% of all sequences. This requirement was then relaxed in two ways: First, using available data that correlated epitope recognition with clinical viral load, we sought to include complete epitopes that were associated with low viral load and exclude epitopes that were associated with high viral load. Secondly, we allowed 1 toggle (variable) site/CE segment if the 2 most common AA at that site are together found in >99% of all known sequences [35]. To accommodate this variation, we created two plasmids, each with 7 CE segments from 12–24 AA in length, separated by 2–4 AA spacers (typically Ala-Ala-X) and differing only by the single toggle AA. The length and sequence of the spacers was set based on the existing knowledge of cleavage specificities and peptide availability [74], as well as to avoid fortuitous junctional homologies to HIV and the human proteome, the latter determined by searching against the HIV and human protein sequence databases.

### DNA plasmids

The *p24CE* and *gag* gene coding sequences were designed by RNA/codon optimization for efficient expression in mammalian cells [60]–[63] and chemically synthesized (GeneArt, Life Technologies, Grand Island, NY). The genes were cloned into the pCMVkan vector [64] optimized for high gene expression. pCMVkan contains the human cytomegalovirus promoter, and the expressed transcripts contain a optimal surrounding for the AUG initiator codon from HIV-1 *tat* that prevents initiation of translation from internal AUGs [75], the bovine growth hormone (BGH) polyadenylation site, and the kanamycin resistance gene. This vector does not contain any splice sites or introns. The p24CE1 and p24CE2 proteins were produced from independent vectors (plasmids 164H and 182H, respectively). The secreted forms SP-p24CE1 and SP-p24CE2 contain the GM-CSF signal peptide (AA 1-17; Genbank accession Nr. NP_000749) at the N terminus (plasmids 234H and 235H). The MCP3-p24CE1 and MCP3-p24CE2 (plasmids 167H and 201H) are fusion proteins with the monocyte chemoattractant protein 3 (MCP-3) [64]-[66]. The LAMP-p24CE1 and LAMP-p24CE2 (plasmids 191H and 202H) are fusion proteins with the lysosomal associated membrane protein 1 (LAMP-1) [67]-[70]. Full-length p55^gag^ proteins were produced from RNA/codon optimized genes cloned into the pCMVkan plasmid, expressing Gag from clade A (plasmid 187H, Genbank accession number AAQ98129), clade B (plasmid 114H, HXB2, Genbank accession number AAB50258), clade C (plasmid 160H, Genbank accession number AAD12096) and the center-of-tree COT-M (222H) [76]. For immunizations HXB2 p55^gag^ was used. Endotoxin-free DNAs were prepared using Qiagen kit according to the manufacturer's protocol (Qiagen, Valencia, CA)

### Transfection and protein analysis

DNA plasmids were transfected into 1×10^6^ HEK-293 cells using the calcium phosphate co-precipitation technique. Culture supernatants and cells were harvested 24 or 48 hours later, and protein expression was visualized by Western immunoblot analysis. The proteins were resolved on 10% or 12% NuPAGE Bis-Tris gels (Invitrogen, Carlsbad, CA), transferred onto nitrocellulose membranes (Invitrogen), which were probed with a goat anti-p24^gag^ antibody (dilution 1∶3000, provided by L. Arthur, SAIC, NCI, Frederick) followed by anti-goat IgG-HRP labeled antibody (dilution 1∶10,000; Calbiochem, EMD chemicals, Gibbstown, NJ) or with plasma (1∶200 dilution) from DNA vaccinated mice followed by anti-mouse IgG-HRP labeled (1∶10,000 dilution, GE Healthcare, Piscataway, NJ). As control, the membranes were probed with anti-human pan-actin antibody (clone C4, EMD Millipore, Billerica, MA) at a dilution of 1∶10,000. The bands were visualized using the enhanced chemiluminescence (ECL) plus Western blotting detection system (GE HealthCare, Piscataway, NJ).

### Mouse DNA vaccination studies

Female C57BL/6N (6 to 8 weeks old) were obtained from Charles River Laboratories, Inc. (Frederick, MD) and were housed at the National Cancer Institute, Frederick, MD, in a temperature-controlled, light-cycled facility. The mice were immunized with 20 µg of the vaccine DNAs by intramuscular injection followed by *in vivo* electroporation by ELGEN^®^ constant current electroporation device (Inovio Pharmaceuticals, Inc, Blue Bell, PA). As negative controls, a group of mice received equal amount of sham DNA following the same immunization protocol. The animals were vaccinated two (0 and 4 weeks) or three times (0, 3, and 6 weeks), and were sacrificed 2 weeks after the last vaccination when spleens and blood were collected for the analysis of cellular and humoral responses.

### Intracellular cytokine staining

The frequency of antigen specific cytokine^+^ T cells was measured using polychromatic flow cytometry, as previously described [66]. The following set of 15-mer Gag peptide pools, overlapping by 11 AA, were used to stimulate the vaccine-induced cellular responses: HIV-1 consensus clade A (Cat# 8116), consensus clade C (Cat# 8118), and Group M Consensus (Cat# 11057), obtained from the AIDS Research and Reference Reagent Program (Germantown, MD); Gag 15-mer from HXB2/Clade B (Infinity Biotech Research & Resource, Inc., Aston, PA). Peptide pools spanning p55^gag^, p24^gag^ or only CE were generated. In addition, we used pools of 10-mer peptides overlapping by 9 AA from COT-M spanning the individual CE1-CE7, not including linker sequences, of p24CE1 and p24CE2 (peptide synthesis facility of the Massachusetts General Hospital, Boston). Splenocytes were cultured at 37°C and 5% CO_2_ at a density of 2×10^6^ cells/ml in complete RPMI-1640 medium containing Gag peptide pools at a final concentration of 1 µg/ml of each peptide. In all experiments, splenocytes cultured in medium without peptide pools or stimulated with phorbol myristate acetate (PMA) and calcium ionophore (Sigma, St. Louis, MO) were used as negative and positive control respectively. Protein secretion was blocked by the addition of monensin (GolgiStop, BD Biosciences) 1 hour after stimulation. After 12 hours incubation, the cells were harvested and cell surface staining was performed using the following antibody cocktail: CD3-APCCy7, CD4-PerCP, and CD8-Pacific Blue (BD Pharmingen, San Diego, CA). Splenocytes were washed twice, fixed, permeabilized with Cytofix/Cytoperm (BD Pharmingen) and staining for intracellular cytokine detection was performed using IFN-γ-FITC (BD Pharmingen). In another set of experiments, the antibody cocktail for surface staining included: CD3-AF700, CD4-PerCP, CD8-Pacific Blue, CD44-V500, CD62L-PE, CD107a-PE Cy7 (BD Pharmingen). The anti-CD107a antibody was added during the culturing of splenocytes with peptides. IFN-γ-APC and TNF-α-APC Cy7 (BD Pharmingen) were used for intracellular cytokine staining. After intracellular staining, the cells were washed twice and the samples were analyzed on an LSR II flow cytometer (BD Pharmingen). Data analysis was performed using the FlowJo platform (Tree Star, Inc., Ashland, OR). All antigen specific responses are reported after subtracting values obtained from the samples without peptide stimulation. Only splenocytes giving a response more than two fold higher than the value of the sample without peptides (medium alone) were considered positive.

### Antibody assays

Serial dilutions of plasma samples were analyzed by standard HIV-1 clade B p24^gag^ ELISA (Advanced Bioscience Lab, Rockville, MD), measuring optical absorbance at 450 nm.

### Ethics Statement

The animal user protocol was approved by the NCI-Frederick Animal Care and Use Committee (AWA#:A4159-01). Frederick National Laboratory for Cancer Research is accredited by AAALAC International and follows the Public Health Service Policy for the Care and Use of Laboratory Animals.
